# Cortical Auditory Evoked Potentials in Cognitive Impairment and Their Relevance to Hearing Loss: A Systematic Review Highlighting the Evidence Gap

**DOI:** 10.3389/fnins.2021.781322

**Published:** 2021-11-18

**Authors:** Hanne Gommeren, Joyce Bosmans, Emilie Cardon, Griet Mertens, Patrick Cras, Sebastiaan Engelborghs, Angelique Van Ombergen, Annick Gilles, Marc Lammers, Vincent Van Rompaey

**Affiliations:** ^1^Department of Translational Neurosciences, Faculty of Medicine and Health Sciences, University of Antwerp, Antwerp, Belgium; ^2^Department of Otorhinolaryngology and Head and Neck Surgery, Antwerp University Hospital, Edegem, Belgium; ^3^Department of Neurology, Antwerp University Hospital and Institute Born-Bunge, University of Antwerp, Antwerp, Belgium; ^4^Department of Neurology, University Hospital Brussel and Center for Neurosciences (C4N), Vrije Universiteit Brussel (VUB), Brussels, Belgium; ^5^Department of Biomedical Sciences, Institute Born-Bunge, University of Antwerp, Antwerp, Belgium; ^6^Department of Education, Health and Social Work, University College Ghent, Ghent, Belgium

**Keywords:** event related potentials (ERP), cortical auditory evoked potential (CAEP), hearing, vestibular function, dementia, Alzheimer’s disease, mild cognitive impairment

## Abstract

**Background:** Alzheimer’s disease (AD) is the most prevalent cause of dementia which affects a growing number of people worldwide. Early identification of people at risk to develop AD should be prioritized. Hearing loss is considered an independent potentially modifiable risk factor for accelerated cognitive decline and dementia in older adults. The main outcome of interest of this review is the alteration of Cortical Auditory Evoked Potential (CAEP) morphology in an AD or mild cognitive impairment (MCI) population with and without hearing loss.

**Methods:** Two investigators independently and systematically searched publications regarding auditory processing on a cortical level in people with cognitive impairment (MCI or AD) with and without hearing loss. Only articles which mentioned at least one auditory elicited event-related potential (ERP) component and that were written in English or Dutch were included. Animal studies were excluded. No restrictions were imposed regarding publication date. The reference list of potential sources were screened for additional articles.

**Results:** This systematic review found no eligible articles that met all inclusion criteria. Therefore, no results were included, resulting in an empty systematic review.

**Conclusion:** In general, dysfunction – being either from cognitive or auditory origin – reduces CAEP amplitudes and prolongs latencies. Therefore, CAEPs may be a prognostic indicator in the early stages of cognitive decline. However, it remains unclear which CAEP component alteration is due to cognitive impairment, and which is due to hearing loss (or even both). In addition, vestibular dysfunction – associated with hearing loss, cognitive impairment and AD – may also alter CAEP responses. Further CAEP studies are warranted, integrating cognitive, hearing, and vestibular evaluations.

## Introduction

As the world’s population increases in age, a growing number of people are confronted with cognitive impairment and dementia. The most common cause of dementia is Alzheimer’s disease (AD) which, according to the World Health Organization, accounts for up to seventy percent of all dementia cases (World Health Organization, 2019).

When people experience a greater-than-expected cognitive decline but are still able to perform their activities of daily life autonomously, the concept mild cognitive impairment (MCI (Albert et al., 2011; Dubois et al., 2014) is used. When their ability to perform activities of daily life is also impaired, the term dementia is applied (McKhann et al., 2011). When including AD biomarkers in the diagnostic process, the terms prodromal AD or MCI due to AD and dementia due to AD (ADD) should be used instead of the more general terms such as MCI and AD, respectively. For clear writing purposes, for both MCI and prodromal AD the term “MCI” will be used, and both AD and dementia due to AD will be described as “AD.”

The diagnosis of dementia and its subtypes is currently based on patients’ medical history, physical examination, neuropsychological assessment, functioning in instrumental activities of daily life, and biomarkers. Biomarkers include (regional and global) atrophy on brain Magnetic Resonance Imaging (MRI) scan, brain fluorodeoxyglucose (FDG) positron emission tomography (PET) (FDG-PET), cerebrospinal fluid (CSF) biomarkers and amyloid PET. With recent advances in AD biomarkers, earlier and more accurate identification of individuals who are in a prodromal stage of AD has become possible. Early identification of AD is important as therapeutic interventions work best if started at the early stages of cognitive decline (Scharre, 2019).

Regarding the early detection of cognitive decline and the evaluation of conversion from MCI to AD, other parameters may be of added value (Jiang et al., 2015). One promising objective parameter is the measurement of cortical auditory evoked potentials (CAEPs). These CAEPs measure central auditory processing and have already demonstrated their usefulness in the objective evaluation of sound processing up to the level of the central auditory nervous system. Moreover, CAEPs are non-invasive, cheap and free from cultural and educational influences and can therefore aid in the standardization of the diagnostic process of AD (Visram et al., 2015). This is a great advantage in comparison with the neuropsychological assessments, which often rely on a person’s language capacities and therefore may be influenced by a language barrier, cultural differences or hearing impairment.

Assessment of electroencephalography-derived CAEPs provide insight into the extent to which hearing function is preserved on a cortical level. However, CAEP morphology is modulated by dysfunction such as hearing loss and/or cognitive impairment. Hearing loss, affecting a third of the older population, is a potentially modifiable risk factor for dementia (Livingston et al., 2020). Both hearing loss and AD alter CAEP morphology in a partially similar way: they reduce certain CAEP amplitudes and prolong latencies. Therefore it is of importance to untangle which CAEP components are affected by hearing loss and/or which by AD and to what extent. This may aid in the early identification of people at risk for cognitive decline.

## Review Question

Since hearing loss is a potentially modifiable risk factor of cognitive decline within AD, and hearing loss and AD both alter CAEP morphology in a similar way, how are CAEPs affected in people with cognitive impairment (MCI or AD) with or without hearing loss?

## Materials and Methods

The Preferred Reporting Items for Systematic Review and Meta-Analysis (PRISMA) guidelines were adhered for conducting and reporting this systematic review (Page et al., 2021). The protocol was registered at the PROSPERO international prospective register of systematic reviews (PROSPERO ID: CRD42021272589).

### Eligibility Criteria

Participants: Older adults with diagnosed MCI or AD will have to be subdivided in a group with and a group without hearing loss. If possible, but not mandatory, these groups will have to be compared with older adults with preserved cognition. Animal studies will be excluded.

Concept: How are CAEPs affected in different populations: in particular in people with MCI or AD. Therefore, only articles which mention at least one auditory elicited ERP component will be included. Since hearing loss is a recognized potentially modifiable risk factor for increased cognitive decline within AD (Livingston et al., 2020), the impact of hearing loss on CAEPs in people with cognitive impairment (MCI or AD) will be reviewed. The main outcome of interest of this review is the alteration of CAEP morphology in these populations.

Types of sources: This systematic review will consider analytical observational studies including prospective and retrospective cohort studies, case-control studies, and analytical cross-sectional studies. In addition, this systematic review will consider descriptive observational study designs such as descriptive cross-sectional studies. Systematic reviews that meet the inclusion criteria will also be considered.

### Search Strategy

The search strategy was defined and ran in the databases of PubMed, Cochrane, Web of Science, and Scopus. The following search string was used: [(Cortical Auditory Evoked Potential) OR (ERP) OR (CAEP)] AND [(Alzheimer’s disease) OR [“mild cognitive impairment”) OR (MCI)] AND (hearing). The search strategy, including all identified keywords and index terms, were adapted for each included database. The reference list of potential sources were screened for additional articles. Studies published in English and Dutch were included. No restrictions were imposed regarding publication date. The date of the last search was 9 August 2021.

### Study Selection

**FIGURE 1:**
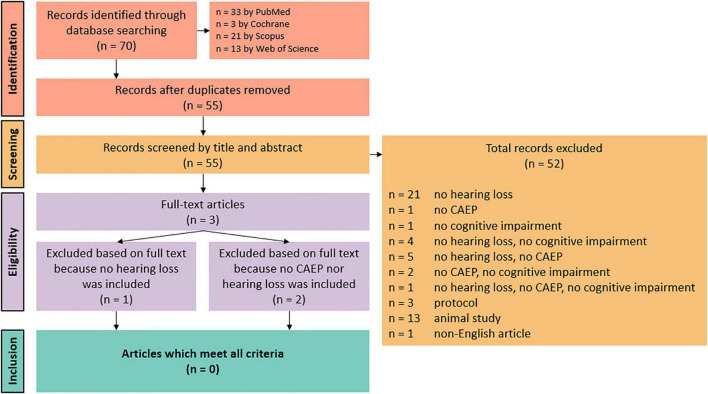
Flow diagram of study inclusion. CAEP, cortical auditory evoked potential.

Following the search, all identified results were collated and uploaded into EndNote X9.2 (Clarivate Analytics, PA, United States). Duplicates were removed. Title and abstracts were then screened by two independent reviewers (HG and JB). Potentially relevant sources were retrieved in full and the full text of selected citations were assessed in detail against the inclusion criteria by two independent reviewers (HG and JB). Discrepancies were discussed until consensus was reached. All steps of the screening procedure are presented in Figure 1, a Preferred Reporting Items for Systematic Reviews and Meta-analyses flow diagram (Page et al., 2021).

## Results

A total of 70 records were identified through database searches. There were no records identified through other sources. After duplicates removal, 55 records were identified. Based on title and abstract, 52 records were excluded because they did not include at least one auditory elicited ERP component, and/or they did not include people with cognitive impairment (either MCI or AD), and/or they did not include people with and without hearing loss. Other reasons for exclusion based on title and abstract were protocols, animal studies, and non-English or non-Dutch articles. The full texts of the remaining three articles were obtained, but these records also did not meet all inclusion criteria. One article did not include people with and without hearing loss, and two articles did not include at least one auditory elicited ERP component nor include people with and without hearing loss. Thus, no results were included in this systematic review. A summary of the inclusion process can be observed in Figure 1.

Even though no record met all inclusion criteria, they have useful information regarding auditory processing on a cortical level in people with cognitive impairment (MCI or AD), or in people with hearing loss.

### Supplementary Findings

An electroencephalography-derived (EEG) auditory evoked potential (AEP) is elicited when acoustic stimuli are presented to a patient. This AEP consists of multiple peaks, each with a certain amplitude (μV), representing the strength of the response, and latency (ms), being the time after stimulus onset. The presence, amplitude, and latency of each peak are determined by the intactness and functionality of the auditory pathway, and properties of the acoustic stimulus (Cone-Wesson and Wunderlich, 2003). Based on their latencies, AEPs can be categorized into three classes (Picton et al., 1974). Auditory brainstem responses (ABRs) (0–8 ms after stimulus onset) and middle latency AEPs (8–50 ms after stimulus onset) are generated by the transmission of sound from the cochlea to the upper brainstem and subcortical areas (Naatanen and Teder, 1991). Auditory late-latency responses (≥50 ms after stimulus onset) represent the transitional process from auditory sensation to conscious perception by widespread activation of the cortex (Joos et al., 2014). Hence, the term cortical auditory evoked potentials (CAEPs) is fit since higher-order auditory-cognitive processing is involved. CAEPs include P50, P100 (P1), N100 (N1), P200 (P2), N200 (N2), Mismatch Negativity (MMN), and P300 (P3).

### Cortical Auditory Evoked Potentials in Hearing Loss

Hearing loss, gradually raising in prevalence and severity with age, has been recognized as one of the most important potentially modifiable risk factors for dementia (Gallacher et al., 2012; Scholes and Mindell, 2014; Livingston et al., 2017). In order to assess to which extent hearing function is preserved on a cortical level, CAEPs can provide an objective measure.

Present P100-N100-P200 peaks indicate intact sound detection because auditory processing at the auditory cortex is maintained. In contrast, the preconscious MMN and conscious N200 and P300 reflect whether the brain can discriminate between different speech stimuli (Naatanen et al., 1993). The MMN, which is a negative wave in the latency window of approximately 100 to 300 ms, is calculated by subtracting event-related potential responses to the standard stimuli from those to the deviant in an oddball paradigm (Kim, 2015).

Sensorineural hearing loss, hearing loss caused by damage to the inner ear or vestibulocochlear nerve, modulates obligatory P100-N100-P200 responses, resulting in prolonged latencies and reduced amplitudes (Adler and Adler, 1989; Oates et al., 2002). McClannahan et al. (2019) found prolonged P100-N100-P200 responses in older adults (62 to 84 years) with age-related hearing loss (ARHL) in comparison with older, normal hearing adults. However, this prolongation was only present at equal sound pressure levels between groups. When equal sensation level stimuli were applied, the prolongation disappeared and only enhanced N100 amplitudes were observed in older adults with ARHL (McClannahan et al., 2019). Enlarged N100 amplitudes have previously been associated with diminished central inhibition (Tremblay et al., 2003).

Oates et al. (2002) analyzed N100, MMN, P200, and P300 responses to speech stimuli (/ba/and/da/) during an auditory oddball paradigm in adults with and without sensorineural hearing impairment (Oates et al., 2002; McClannahan et al., 2019). In this study, more severe degrees of hearing loss were associated with prolonged latencies and decreased amplitudes, up to the point where waveforms disappeared completely. A note of caution is due here since equal sound pressure levels were used, suggesting that these findings represent audibility rather than the degree of hearing loss, as previously argued by McClannahan et al. (2019).

In general, latency measures are more sensitive in detecting changes in hearing function, whereas amplitudes are more dependent on the person’s attention as well as stimulus properties, such as stimulus intensity (Pratt et al., 2009; Vonck et al., 2019). This is especially so for later CAEPs (N200 and P300), suggesting an earlier impact of hearing loss on higher-order (non-sensory) cortical processing, and later on lower-order (sensory) cortical processing (N100 and MMN) (Oates et al., 2002).

When presenting acoustic stimuli to people with (potential) hearing loss, attention must be paid to the sound level of these stimuli. In general, prolonged latencies and reduced amplitudes are found in subjects with hearing loss in comparison with people with normal hearing when using stimuli at equal sound pressure levels (Adler and Adler, 1989; Oates et al., 2002). A similar pattern is found when the stimulus intensity approaches the hearing threshold (Lightfoot and Kennedy, 2006). Therefore, signal attenuation, due to either hearing loss (with stimuli at equal sound pressure levels) or reduced stimulus intensity, modulates obligatory CAEP responses. These results may reflect audibility rather than the actual impact of hearing loss on CAEP components and subsequent auditory processing (McClannahan et al., 2019). Future studies controlling for audibility, either by using stimuli at equal sensation levels or by using control groups matched at their hearing level, are therefore recommended. An overview of the most relevant findings is listed in Table 1.

**TABLE 1:** Overview of the most relevant findings concerning CAEPs in hearing loss and Alzheimer’s disease.

	**Author (year)**	**CAEP component**	**Amplitude**	**Latency**	**Additional findings**
Hearing loss	Adler and Adler (1989)	P100-N100-P200	Reduced	Prolonged	
	Oates et al. (2002)	P100-N100-P200	Reduced	Prolonged	More severe degrees of hearing loss are associated with prolonged latencies and decreased amplitudes
	Tremblay et al. (2003)	N100	Enhanced		
	Lightfoot and Kennedy (2006)	P100-N100-P200	Reduced	Prolonged	
	McClannahan et al. (2019)	P100-N100-P200		Prolonged	
		N100	Enhanced	Prolonged	
Alzheimer’s disease	Jiang et al. (2015) *	P300	Reduced	Prolonged	More severe degrees of cognitive impairment are associated with more prolonged latencies
	Lister et al. (2016)	P200	Reduced		
	Gu and Zhang (2017) *	P200		Prolonged	More severe degrees of cognitive impairment are associated with more prolonged latencies
		N200		Prolonged	
		P300	Reduced	Prolonged	

CAEP, cortical auditory evoked potential. *Asterisks indicate results from a meta-analysis.

### Cortical Auditory Evoked Potentials in Alzheimer’s Disease

**FIGURE 2:**
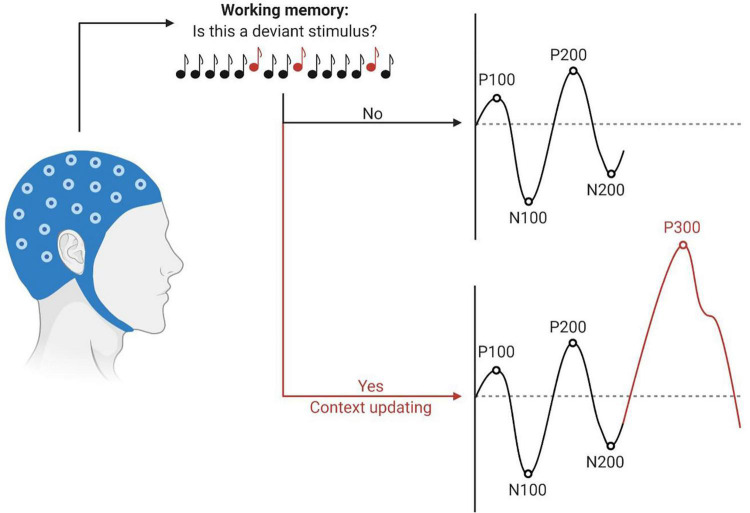
Schematic illustration of the P300 context-updating model (Polich, 2003). Participants undergo an auditory oddball task during EEG recording. Stimuli are processed and evaluated in the working memory. If the stimulus is identical to the previous one, the mental model remains unchanged, and obligatory responses are evoked (P100, N100, P200, N200). If the stimulus deviates from the standard and participants have allocated their attention to the target stimulus, the mental model of the stimulus environment is updated. The P300 response is elicited in addition to the sensory evoked potentials. Latencies are depicted on the *x*-axes, amplitudes on the *y*-axes. This figure has been generated using BioRender^®^.

As dementia alters cognitive processing in the brain, the characteristics of certain higher-order CAEPs, may be affected. In particular, the P300, or P3, component is a positive wave generally occurring around 300 ms post-stimulus. It is elicited by an oddball paradigm, in which rare “target” or “deviant” stimuli are randomly embedded in a train of often-recurring “standard” stimuli. The P300 component is evoked only by deviant stimuli and requires attention towards the stimuli, suggesting a top-down process, as depicted in Figure 2 (Picton, 1992; Linden, 2005). Latencies of the P300 component reflect neural speed, whereas P300 amplitudes index cognitive resources (van Dinteren et al., 2014). Jiang et al. (2015) performed a meta-analysis on studies measuring the P300 component in patients with MCI and AD. They found an increasingly more significant prolongation of P300 latency in individuals who were more impaired along the AD continuum. In addition, P300 amplitude was significantly smaller in the cognitively impaired group in comparison with older adults with age-normal cognition. An altered P300 morphology implies an increased processing time and further impairment of stimulus classification and cognitive functioning in general. Hence, the P300 component and in particular its latency, may be a sensitive early-stage diagnostic marker for cognitive decline. Furthermore, it may be an indicator of disease progression, as individuals who progress from MCI to AD show a more distorted P300 morphology in comparison with stable MCI patients (Jiang et al., 2015). A subsequent meta-analysis by Gu and Zhang (2017) supported previous research. Additionally, it was demonstrated that delayed latencies of the P200 and N200 components could also be potential electrophysiological markers in discriminating people with impaired cognition from those in which cognition is preserved (Gu and Zhang, 2017). Besides, Lister et al. (2016) examined P100-N100-P200 CAEP responses using a passive auditory paradigm and found smaller P200 amplitudes in people with probable MCI compared to cognitively normal older adults. An overview of the most relevant findings is listed in Table 1.

Thus, both cognitive impairment (MCI and AD) and hearing loss may alter CAEP’s waveform morphology and should be integrated in future research when assessing CAEP measurements. Research that incorporates the role of hearing function on CAEP potentials in determining the risk of developing dementia is currently lacking.

As previously stated, hearing loss is a potentially modifiable risk factor for cognitive decline and dementia, and impacts CAEP morphology. However, the other sensory organ in the inner ear, being the vestibular apparatus, may also be associated with cognitive decline and may also alter CAEP morphology.

## Discussion

This study aimed to systematically review the literature on how CAEPs are affected in people with cognitive impairment (MCI or AD) with and without hearing loss. Unfortunately, the search resulted in no included studies reporting both the evaluation of hearing and cognitive status in CAEPs, resulting in an empty systematic review. Nevertheless, CAEPs can be a valuable measurement in the assessment of both hearing loss and cognitive decline.

Because of their close anatomical relationship, there is a high occurrence of hearing loss in patients with vestibular dysfunction and vice versa. Early in evolution, otolith organs encompassed vestibular function, as well as sound detection. As vertebrates evolved, the cochlea became mainly responsible for sound detection (Fay and Popper, 1999). Nevertheless, the otolith organs maintained some acoustic sensitivity (as can currently be observed by vestibular side effects such as nystagmus and evoked electromyographic signals elicited by vestibular evoked myogenic potential (VEMP) testing) (Lackner and Graybiel, 1974; Rosengren et al., 2019).

Vestibular function is important in the reflexive coordination of eye, head, and body movement in a three-dimensional space, voluntary movement and balance, and higher cognitive functions such as navigation and spatial memory. Typical symptoms of vestibular dysfunction include unsteadiness, dizziness, disorientation, and vertigo. Although intact vestibular functioning is crucial for normal movement and overall quality of life, it is one of the most often overlooked sensory systems in EEG research (Dieterich and Brandt, 2015; Agrawal et al., 2017).

Besides hearing loss, vestibular dysfunction, affecting one in every five older adults, is also associated with cognitive impairment and AD and may even contribute to its onset (Previc, 2013; Sogebi et al., 2014; Harun et al., 2016). For a review on this topic, see Bosmans et al. (2020).

To the best of our knowledge, only one recent study evaluated CAEP recordings in individuals with peripheral vestibular dysfunction. Santos Filha et al. (2018) performed the auditory oddball paradigm in nineteen adults (age 20 to 80 years old) with vestibular dysfunction. Regarding hearing function, people with an average hearing threshold up to 25 dB HL in the low frequencies (from 250 to 2,000 Hz) and up to 50 dB HL in the high frequencies (from 3,000 to 8,000 Hz) were included. They demonstrated increased latencies in CAEP components (P100, N100, P200, N200, P300) in adults with vestibular dysfunction. However, these delayed latencies were not statistically significant (Santos Filha et al., 2018). Studies with bigger sample sizes may provide more substantiated insights into the possible alterations of CAEP components in people with vestibular dysfunction.

In conclusion, vestibular dysfunction may alter CAEP’s waveform morphology and is associated with cognitive impairment and AD. However, literature that incorporates vestibular evaluation, CAEP potentials and dementia is currently unavailable.

### Future Directions

In general, decreased CAEP amplitudes and prolonged latencies indicate malfunctioning, which may involve a peripheral problem such as hearing loss, or a central problem such as cognitive impairment. Hence, people with AD or hearing loss may show a similar pattern of distorted CAEP waveform morphologies. It is therefore important to incorporate hearing assessments when evaluating CAEP measurements in people with AD, and likewise incorporate cognitive assessments when evaluating CAEP measurements in people with hearing loss. That way, the malfunctioning (being either hearing loss, cognitive decline, or both) associated with distorted CAEP waveform morphologies can be assessed more accurately.

In addition to integrating hearing and cognitive assessments when evaluating CAEP measurements, it is also important to include vestibular function tests. Not only are the auditory and vestibular end organs anatomically nearby and closely intertwined, there is a high occurrence of vestibular loss in people with hearing loss and vice versa. Additionally, vestibular dysfunction has demonstrated to prolong CAEP latencies, despite not reaching statistical significance (Santos Filha et al., 2018). Furthermore, as CAEPs assess the integrity of the auditory pathway from the peripheral end organ to the auditory cortex, and since vestibular disorders can distort those components of the auditory pathway, the integration of vestibular testing in CAEP research is recommended (Santos Filha et al., 2018). Overall, in CAEP research, there is limited literature available that examines the exact contributions of vestibular function, let alone vestibular dysfunction. This emphasizes the need for further research, ideally with larger patient groups, in order to clarify the link between vestibular (dys)function and CAEPs.

## Conclusion

To the best of our knowledge, this is the first attempt to systematically review how CAEP morphology is affected in people with cognitive impairment (MCI or AD) with or without hearing loss. The results of our systematic search for literature concerning both the impact of hearing and cognitive status on CAEPs revealed that there are currently no published studies exploring this specific topic. Therefore, this systematic review increases the awareness of an “evidence gap” in this field of research, especially because hearing loss is a potentially modifiable risk factor for an accelerated cognitive decline and modulates CAEP responses.

In conclusion, distorted waveform morphologies of CAEPs may serve as (early) prognostic indicators of cognitive decline and disease progression along the AD continuum. CAEPs have the advantage of being non-invasive, objective, and applicable in case of language barrier (Jiang et al., 2015; Gu and Zhang, 2017). Early identification of people affected with cognitive decline and those at risk for it is crucial, as it will aid in providing therapeutic interventions to those who will benefit most from it at the very early stages of cognitive decline. Non-pharmacologic interventions may include cognitive therapy, physical exercise, occupational therapy, emotional and social stimulation, etc., (IQWiG, 2006). These interventions aim to improve a patient’s functional abilities and coping strategies to maximize the quality of life. The goal is to maintain the intact cognitive capacities and compensate for those that have deteriorated (Mitra and Dey, 2013). In the early stages of cognitive decline, interventions will be able to have the most impact in slowing down further cognitive impairment and progression of dementia in general (Scharre, 2019). Furthermore, regarding the associated vestibular decline with cognitive impairment, early (vestibular) interventions may reduce the risk of unsteadiness and falls in this, already frail, population (Harun et al., 2016; Whitney et al., 2016; Gandhi et al., 2020). With successful interventions, the highest possible degree of quality of life and self-sustainability can be maintained for as long as possible, reducing the burden of AD. In conclusion, further CAEP studies are warranted, integrating cognitive, hearing, and vestibular evaluations.

## Data Availability Statement

The original contributions presented in the study are included in the article/supplementary material, further inquiries can be directed to the corresponding author.

## Author Contributions

VVR, HG, and JB conceived and designed the study. HG and JB drafted the manuscript. All authors critically revised the manuscript for important intellectual content, read, and approved the final manuscript.

## Conflict of Interest

The authors declare that the research was conducted in the absence of any commercial or financial relationships that could be construed as a potential conflict of interest.

## Publisher’s Note

All claims expressed in this article are solely those of the authors and do not necessarily represent those of their affiliated organizations, or those of the publisher, the editors and the reviewers. Any product that may be evaluated in this article, or claim that may be made by its manufacturer, is not guaranteed or endorsed by the publisher.
